# The transcription factor Ste12-like increases the mycelial abiotic stress tolerance and regulates the fruiting body development of *Flammulina filiformis*

**DOI:** 10.3389/fmicb.2023.1139679

**Published:** 2023-05-04

**Authors:** Xiaomeng Lyu, Qingji Wang, Ao Liu, Fang Liu, Li Meng, Panmeng Wang, Yan Zhang, Li Wang, Zhuang Li, Wei Wang

**Affiliations:** ^1^Shandong Provincial Key Laboratory of Agricultural Microbiology, College of Plant Protection, Shandong Agricultural University, Tai’an, China; ^2^Mycological Research Center, College of Life Sciences, Fujian Agriculture and Forestry University, Fuzhou, China

**Keywords:** winter mushroom, pheromone signaling pathway, MAPK, transcription factor, abiotic stress

## Abstract

**Introduction:**

*Flammulina filiformis* is one of the most commercially important edible fungi worldwide, with its nutritional value and medicinal properties. It becomes a good model species to study the tolerance of abiotic stress during mycelia growth in edible mushroom cultivation. Transcription factor Ste12 has been reported to be involved in the regulation of stress tolerance and sexual reproduction in fungi.

**Methods:**

In this study, identification and phylogenetic analysis of *ste12-like* was performed by bioinformatics methods. Four *ste12-like* overexpression transformants of *F. filiformis* were constructed by Agrobacterium *tumefaciens*-mediated transformation.

**Results and Discussion:**

Phylogenetic analysis showed that Ste12-like contained conserved amino acid sequences. All the overexpression transformants were more tolerant to salt stress, cold stress and oxidative stress than wild-type strains. In the fruiting experiment, the number of fruiting bodies of overexpression transformants increased compared with wild-type strains, but the growth rate of stipes slowed down. It suggested that gene *ste12-like* was involved in the regulation of abiotic stress tolerance and fruiting body development in *F. filiformis*.

## 1. Introduction

*Flammulina filiformis* (previously named *Flammulina velutipes*), also known as the Winter Mushroom or Enokitake, is an important edible and medicinal mushroom that is cultivated on a large scale (Wang et al., 2012; Zhang et al., 2012; Tao Q. et al., 2016; Dai and Yang, 2018; Wang P. M. et al., 2018). It is also one of the mushrooms with industrial cultivation at present (Wang Q. Y. et al., 2018). In the process of industrial cultivation of mushrooms, strict clean environment control greatly reduces the occurrence of pests and diseases. Understanding the response mechanism of abiotic stress will help us to obtain better-cultivated varieties to cope with the adverse environment and achieve efficient production of edible fungi. Thereby, the effect of abiotic stress during the mycelial culture process and the fruiting body development become major scientific issues. *F. filiformis* has stable cultivation characteristics and mature genetic operation technology, which can be used as a potential model material for studying the growth and development regulation mechanism of mushrooms (Park et al., 2014).

The frequently conducted studies concerning abiotic stress tolerance are necessary and generally interesting topics in fungi biology. Melatonin existed extensively in mushrooms and enhanced cadmium tolerance through antioxidant-related metabolites and enzymes. It relieved Cd-induced damage in the *Volvariella volvacea* (Gao et al., 2020). The expression levels of genes related to carotene production under oxidative and osmotic stress were studied by quantitative real-time PCR (qRT-PCR) in *Cordyceps militaris*. It was reported that *C. militaris* could produce a large amount of glycerol and carotenoids to resist high oxidative stress when cultured in a saline–alkali environment for a long time (Zhao et al., 2021). In the edible mushroom, members of the C2H2-type zinc finger (C2H2 Znf) transcription factor expression levels are changed suddenly under heat and cold stress, suggesting that these genes may participate in abiotic stress responses (Ding et al., 2022).

The MAPK signaling pathway widely exists in various eukaryotes and is involved in cell division, differentiation, apoptosis, and other life processes (Mu and Chen, 2002; Hagiwara et al., 2016). The pheromone signaling pathway is one of the MAPK signaling pathways (Tatjer et al., 2016; Li et al., 2017; Deng and Lin, 2018). It was reported that the pathway participated in the mating, growth and development, and morphogenesis of yeast and filamentous fungi (mostly pathogenic fungi) (Cervantes-Chávez and Ruiz-Herrera, 2006; Hoi and Dumas, 2010; Palacios et al., 2011; Takemoto et al., 2011; Kitade et al., 2015; Leng and Song, 2016; Chen et al., 2019). After mating, the pheromone signaling pathway is “turned on” by mating type *B* genes in the dikaryotic mycelia, and transcription factor Ste12 is activated (Brown and Casselton, 2001). The *Saccharomyces cerevisiae* Ste12 protein regulates the traits of mating and invasion by interacting with other transcription factors, to bind and activate distinct sets of genes in response to mating pheromones or nutrients, respectively (Zhou et al., 2020). The first Ste12 gene was isolated from the yeast *S. cerevisiae* (Errede and Ammerer, 1989). After this, other homologous transcription factors that contain two C-terminally located tightly linked C2H2 Znf were named Ste12-like factors (Hoi and Dumas, 2010; Wei et al., 2017). The Ste12-like transcription factor MaSte12 is involved in the pathogenicity by regulating the appressorium formation in *Metarhizium acridum* (Wei et al., 2017). Homeodomain transcription factor Ste12 is involved in the virulence and pathogenicity of filamentous fungi (Schamber et al., 2010; Wei et al., 2017; Xu et al., 2018; Zhu et al., 2018; Liu et al., 2020; Lin et al., 2021). Yeast transcription factor Ste12 plays a role in response to osmotic, high temperature, low pH, starvation, and other stress (Morillon et al., 2000; Gancedo, 2001; Morishita et al., 2002; Heise et al., 2010; Schamber et al., 2010; Vidal et al., 2013; Zhou et al., 2020; Purohit and Gajjar, 2022). The transcription factors in shared orthogroups included the light-sensing white collar complex member WC-1, orthologs of *S. cerevisiae* sexual reproduction-related Ste12, and are important for sexual morphogenesis (Merényi et al., 2022). In addition, the metabolites related to adaptation to environmental changes and stress resistance, such as arginine and proline, are accumulated, so that the dikaryotic mycelium obtained better adaptability to environmental stress (Yang and Gao, 2007; Wang et al., 2015).

The gene *ste12-like* encoding a dual C2H2 Znf transcription factor domain is located downstream of the pheromone signaling pathway and is a key factor in fungal growth and development (Hoi and Dumas, 2010; Steindorff et al., 2022). It is involved in the regulation of sexual reproduction, the pathogenicity of most pathogenic fungi, and osmotic stress (Bardwell et al., 1998; Vallim et al., 2000; Kim et al., 2009; Wilson et al., 2022). Three downstream MAPK pathway TFs (Rlm1, Swi6, and Ste12) of MAPK pathways have been demonstrated to contribute to the stress response and found to be involved in the pathogenesis of *Fusarium oxysporum* (Zuriegat et al., 2021). In our previous study, pheromone signaling pathway elements were annotated, and the *ste12-like* was a differentially expressed gene in the fruiting body development of *F. filiformis*. The expression of gene *ste12-like* was down-regulated in elongation stipe. It suggested that the overexpression of *ste12-like* might inhibit the elongation of the stipe (Liu et al., 2022). In this study, the function of transcription factor encoding gene *ste12-like* in the mycelial abiotic stress and fruiting body development in *F. filiformis* was studied. The results could help to further analyze fungal abiotic stress tolerance and fruiting body development regulation.

## 2. Materials and methods

### 2.1. Strains and media

The *F. filiformis* dikaryotic strain FL19 was the receptor strain of overexpression. The *F. filiformis* monokaryotic strain L11 (Protoplast mononuclear strain from FL19) was used for cloning gene *ste12-like*. The strain was maintained on potato dextrose agar medium (PDA; 200 g/L of potato; 20 g/L of glucose; 20 g/L of agar; Solarbio, China) at 25°C and provided by the Fujian Edible Fungi Germplasm Resource Collection Center of China. *Escherichia coli* strain DH5α (Vazyme, China) was used for cloning and plasmids propagation, while *Agrobacterium tumefaciens* strain GV3101 (TransGen Biotech, China) was used for transferring the plasmids into *F. filiformis*.

Induction medium (IM) included 10 mM glucose, 10 mM K_2_HPO_4_, 10 mM KH_2_PO_4_, 0.7 mM CaCl_2_, 2 mM MgSO_4_·7H_2_O, 9 μM FeSO_4_·7H_2_O, 2.5 mM NaCl, 4 mM (NH_4_)_2_SO_4_, 0.5%(w/v) glycerol, 200 μM acetosyringone (AS), and 40 mM 2-(N-Morpholino)ethanesulfonic acid (MES) (pH5.3) (Nielsen and Sørensen, 1997; Shi et al., 2012; Du et al., 2022).

To produce fruiting bodies, strains were cultivated in tissue culture bottles containing growth substrate (cottonseed hulls of 53.5%, wheat bran of 25%, sawdust of 20%, gypsum of 1%, and ground limestone of 0.5%, with a water content of 60%).

### 2.2. Identification and phylogenetic analysis of gene *ste12-like*

The sequence of *ste12-like* (ID: *gene186*) was obtained from the genome of *F. filiformis* monokaryotic strain L11 (BioProject: PRJNA191865). The sequences of nucleotide and protein of *ste12-like* can be found in the GenBank with accession Nos. OM816714 and UPT49966.1. Nuclear localization signals (NLSs) and protein domains were predicted by online software PSORT II Prediction (^1^Horton et al., 2007) and InterPro 91.0 (^2^Blum et al., 2020). Multiple sequence alignment of the *F. filiformis* Ste12-like and homologous proteins downloading from GenBank were performed with Clustal Omega (^3^Sievers and Higgins, 2014).

DNA sequences were edited and aligned with BioEdit v 7.0.9 (Halling, 1999). In the phylogenetic analyses, the STE-like transcription factor domain-containing protein of *Elsinoe fawcettii* (GenBank accession number: ACT65872.1) was chosen as the outgroup. Sequences of other species were downloaded from NCBI with GenBank numbers. Neighbor-Joining (NJ) analysis was conducted using MEGA 7.0 (Kumar et al., 2016). For NJ analysis, all parameters were kept default (Kumar et al., 2012). Motif prediction of all sequences was performed using the MEME-suite website^4^ and default parameters (Bailey et al., 2015).

### 2.3. DNA extraction, plasmid construction, and fungal transformation

Total genomic DNA was extracted from the mycelia of *F. filiformis* strains, grown on cellophane-covered PDA plates at 25°C for 7 days, using EasyPure Plant Genomic DNA Kit (TransGen Biotech, China) according to the manufacturer’s protocol. Isolated DNA was used as a template for PCR amplification. PCR conditions were as follows: 5 min at 94°C, followed by 35 cycles of 45 s at 94°C, 45 s at annealing temperature, 1 min at 72°C, then 10 min at 72°C.

The binary vector pBHg-BCA1 was provided by the Fujian Edible Fungi Germplasm Resource Collection Center of China. It was used to construct the overexpression plasmid of gene *ste12-like* (Lyu et al., 2021). A schematic representation of *ste12-like* overexpression plasmid constructs is shown in Supplementary Figure 1. In the plasmid, the promoter of *glyceraldehyde-3-phosphate dehydrogenase* (P*gpd*) and terminator of T35S were used to control the expression of gene *ste12-like*.

For the construction of overexpression vector *ste12-like*, the full-length fragment of the gene *ste12-like* was amplified from strain L11 DNA using primer pairs (ste12-like F/R) with added *Spe* I (TaKaRa, Japan) and *Apa* I (TaKaRa, Japan) sites (Table 1). The PCR product was digested with *Spe* I and *Apa* I for 60 min at 37°C and then ligated into the pBHg-BCA1 plasmid using T4 ligase for 12 h at 16°C (Vazyme, China). Then the *ste12-like* overexpression plasmid constructs were transformed into *E. coli* DH5α for cloning (Kanamycin, 50 μg/mL), sequencing, and plasmids propagation and then transformed into *A. tumefaciens* GV3101 for fungal transformation (Lyu et al., 2021; Meng et al., 2021).

**Table 1:** List of primers in this study.

Primer	Sequence 5′-3′	Melting temperature (°C)
ste12-like-F	GGACTAGTATGCACCGCGAGGGCTTC	56
ste12-like-R	AGGGCCCTTAAATAAATAATAAGTCGTGTTGG	
GBT-F	CCCAGGCTTTACACTTTAT	50
GBT-R	AGCATTCGCCATTCAG	
Hpt-F	CTATTCCTTTGCCCTCGG	54
Hpt-R	ATGAAAAAGCCTGAACTCACC	
ACTB-F	GATCGTATGCAGAAGGAGTTGACAC	58
ACTB-R	CCACTCTCGTCGTACTCTTGCTTG	
GAPDH-F	CCTCTGCTCACTTGAAGGGT	58
GAPDH-R	GCGTTGGAGATGACTTTGAA	
ste12-like(qRT-PCR)-F	GTGGGTGGACCTGGGATGAC	58
ste12-like(qRT-PCR)-R	ATGCTGCTGGTGGTGCTGAT	

“ACTAGT” underlined was Spe I site; “GGGCCC” underlined was Apa I site.

Hygromycin sensitivity of *F. filiformis* strain FL19 was tested first. The *F. filiformis* strain FL19 was inoculated on PDA (Solarbio, China) with different concentrations (0 μg/mL, 2.5 μg/mL, 5 μg/mL, 7.5 μg/mL, 10 μg/mL, 12.5 μg/mL, and 15 μg/mL) of hygromycin B (Solarbio, China) and incubated at 25°C for 10 days. Overexpression plasmid Ste12-like^OE^ was transformed into *F. filiformis* receptor strain FL19 using the *Agrobacterium tumefaciens*-mediated transformation (ATMT) approach (Lyu et al., 2021; Meng et al., 2021). Mycelia plugs (diameter 6 mm) from the edge of the FL19 colony were transferred into 50 mL centrifuge tubes together with *A. tumefaciens* in liquid IM for 6 h. After inoculation, co-cultures were maintained on the solid IM medium covered with sterile cellophane at 25°C for 3 days. In order to remove the *A. tumefaciens* as cleanly as possible, co-cultures were rinsed in a 50 mL sterile centrifuge tube, which contained 40 mL of sterile water added to 200 μg/mL of cefotaxime. Finally, mycelia plugs were dried with sterile filter paper and then cultured on the PDA medium supplemented with 12.5 μg/ml of hygromycin B and 100 μg/ml of cefotaxime at 25°C.

All putative transformants were first selected on PDA plates containing hygromycin B (12.5 μg/mL) five times to stabilize the genotype for further use. For integration confirmation, the fragment of gene *hygromycin B phosphotransferase* (*Hpt*) was amplified using primers Hpt (Table 1) to confirm the transformant of *F. filiformis*.

### 2.4. RNA extraction and quantitative real-time PCR

RNA was extracted using OMEGA E.Z.N.A.Plant RNA Kit (Omega Bio-tek, United States). Samples were treated with the RNase-free DNase I for 2 min at 42°C to remove potential genomic DNA contamination in the RNA extraction process. The quality and concentration of the RNA were evaluated by agarose gel electrophoresis and NanoDrop ND-1000 Spectrophotometer (NanoDrop Technologies, USA). RNA samples with A260/A280 ratios of 1.9 ~ 2.0 and concentrations that were higher than 500 ng/μL were used for further analysis. cDNA was synthesized using the same concentration (1 μg/μL) of total RNA by TransScript All-in-One First-Strand cDNA Synthesis SuperMix for qPCR Kit (Transgen, Beijing, China) according to the manufacturer’s protocol. The resultant cDNA samples were stored at −80°C.

Reaction mixtures (25 μL volume) for qRT-PCR contained 0.5 μL of 10 μM of each primer, 12.5 μL of 2 × TransStartTM Top Green qPCR SuperMix, 0.5 μL of Passive Reference Dye/PCR Enhancer (50×), 1 μL of cDNA template, and 10 μL of ddH_2_O. Thermal cycling conditions were as follows: 30 s at 94°C, followed by 40 cycles of 5 s at 94°C and 30 s at 60°C. The fragment size of gene *ste12-like* for qRT-PCR was 84 bp. The range of the dissociation ramp from 60°C to 95°C for 6 s that the fluorescence was acquired after the PCR program. The expression level of gene *ste12-like* was analyzed by qRT-PCR using 2^-△△*C*^_T_ method (Livak and Schmittgen, 2001). The qRT-PCR primers of *ste12-like* and reference genes *glyceraldehyde-3-phosphate dehydrogenase* (*GAPDH*) and *β-actin* (*ACTB*) (Tao Y. et al., 2016, 2019; Wu et al., 2019) are listed in Table 1. All the qRT-PCR primers were designed with flanking introns to prevent the amplification of residual genomic DNA. Three technical replicates and three biological replicates were set for each sample.

### 2.5. Resistance tests of transformants to abiotic stress

To investigate the abiotic stress tolerance, the transformants Ste12-like^OE8^, Ste12-like^OE10^, Ste12-like^OE14^, Ste12-like^OE15^, and wild-type strain FL19 of *F. filiformis* were inoculated on the PDA medium and cultured at 25°C for 7 days. Then, the outer part of the colonies was picked as mycelial plugs (diameter 6 mm) for the abiotic stress test. First, the salt stress test was carried out. The mycelial plugs were inoculated on the center of the PDA medium containing different concentrations (0 g/L, 5 g/L, 10 g/L, 15 g/L, 20 g/L, and 25 g/L) of NaCl and KCl, respectively, at 25°C. Second, the temperature stress test was carried out. The mycelial plugs were inoculated on the center of the PDA medium and incubated at 15°C, 20°C, 25°C, and 30°C, respectively. Third, the oxidative stress test was carried out. The mycelial plugs were inoculated on the center of the PDA medium containing different concentrations (0 mmol/L, 5 mmol/L, 10 mmol/L, and 15 mmol/L) of H_2_O_2_ and incubated at 25°C, respectively. From the third day after inoculation, the diameter of the colony was measured by the cross-over method at regular intervals every day. The colony edge was then marked every 24 h in the following 7 days, and the mycelia growth rate was calculated as the average colony extension per day (Wu et al., 2019). This experiment was repeated three times independently.

### 2.6. Fruiting body cultivation and phenotypic analysis of transformants

Strains were grown at 25°C for 30 days. After the mycelium was full for 3 days, the aged mycelium was scraped with a sterilized inoculation shovel and placed in the same incubator. Cold stimulation was performed at 15°C and 90% humidity until the primordia emerged (1 week). Cultures were maintained at a low temperature (15°C and 75% humidity) to allow the full fruiting body development. The primordia appeared on the 36th day. After 1 week, the stipes grew to the bottle mouth, and then the stipe length was measured every 2 days until maturity on the 51st day. Then the number of fruiting bodies was counted according to the number of pilei. The fruiting bodies were dried at 60°C until constant weight, and the dry weight of each bottle was measured.

### 2.7. Statistical analysis

All experiments described in this study were carried out with three independent replicates to ensure that the trends and relationships observed were reproducible. The error bars indicate the standard deviation (SD) from the mean of triplicate samples. Statistical analyses were conducted using SPSS, version 22.0. One-way ANOVA was used to determine statistically significant differences between samples with IBM SPSS Statistics 22.0. Differences between samples were considered statistically significant at *p* < 0.05. Tukey’s *post hoc* test is further used to test the significant difference between any two-treatment means.

## 3. Results

### 3.1. Bioinformatics analysis of gene *ste12-like*

**Figure 1:**
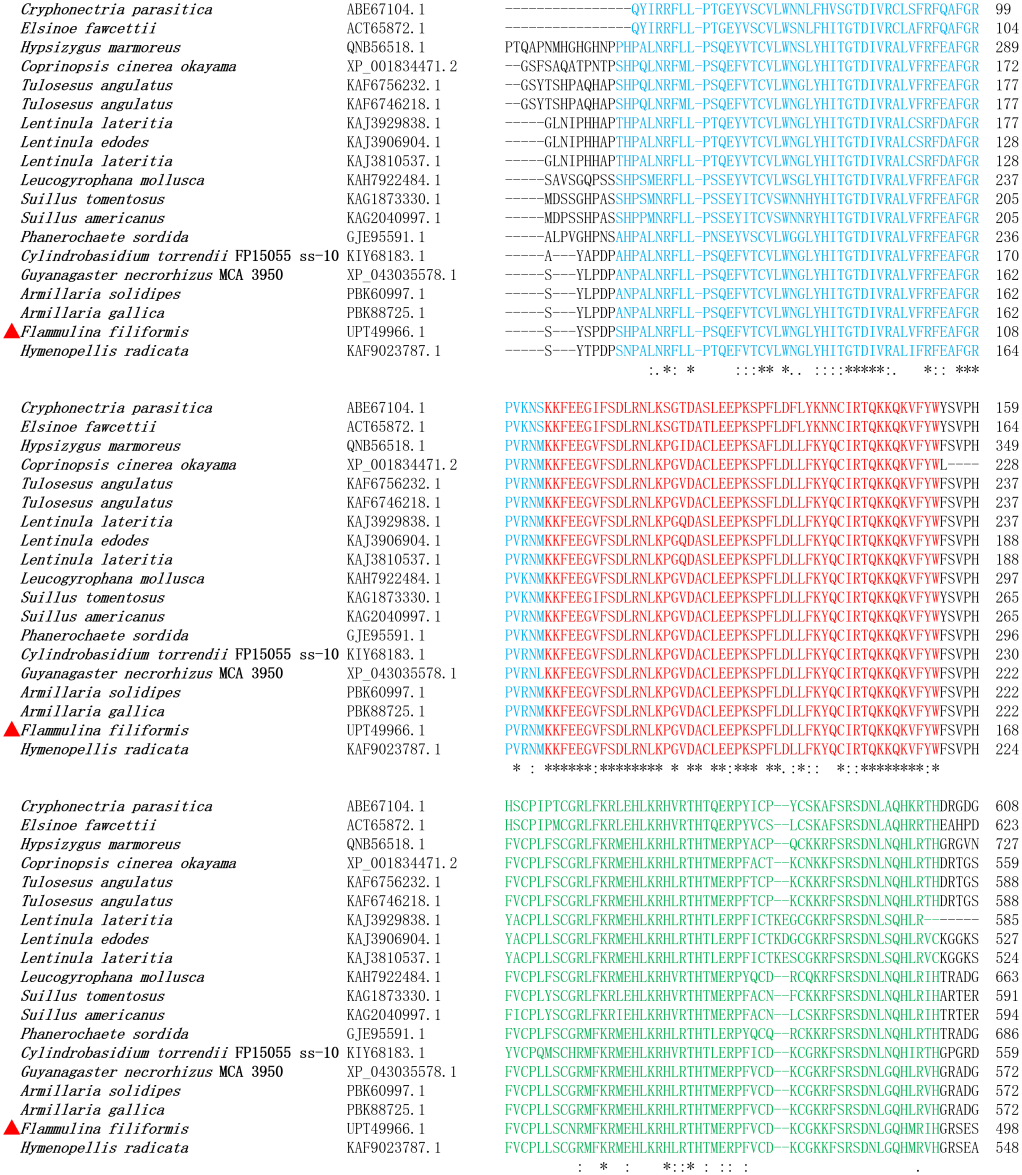
Alignment of *F. filiformis* Ste12-like (GenBank accession No. UPT49966.1 marked in red triangle) and homologous proteins.

The coding sequence of gene *ste12-like* was 2,957 bp, with five exons and four introns, and encodes a protein with 908 amino acid residues. The localization of *F. filiformis* Ste12-like protein was nuclear (Reliability: 89; Reinhardt and Hubbard, 1998) with two NLSs (298–306: PTYKQRRKK; 714–720: PVRRHRS) (Supplementary Table 1) and two DNA-binding motifs (CPLLSCNRMFKRMEHLKRHLRTH; CDKCGKKFSRSDNLGQHMRIH) of Zinc finger, C2H2 type, domain. After protein family prediction, Ste12-like (IPR003120) and C2H2 Znf (IPR036236) families were identified in *F. filiformis* Ste12-like and homologous proteins (Figure 1). Based on bioinformatic analyses, the two NLSs, Ste12-like (IPR003120) and C2H2 Znf (IPR036236) families, with two DNA-binding motifs predicted in Ste12-like revealed that it was a fungal transcription factor.

**Figure 2:**
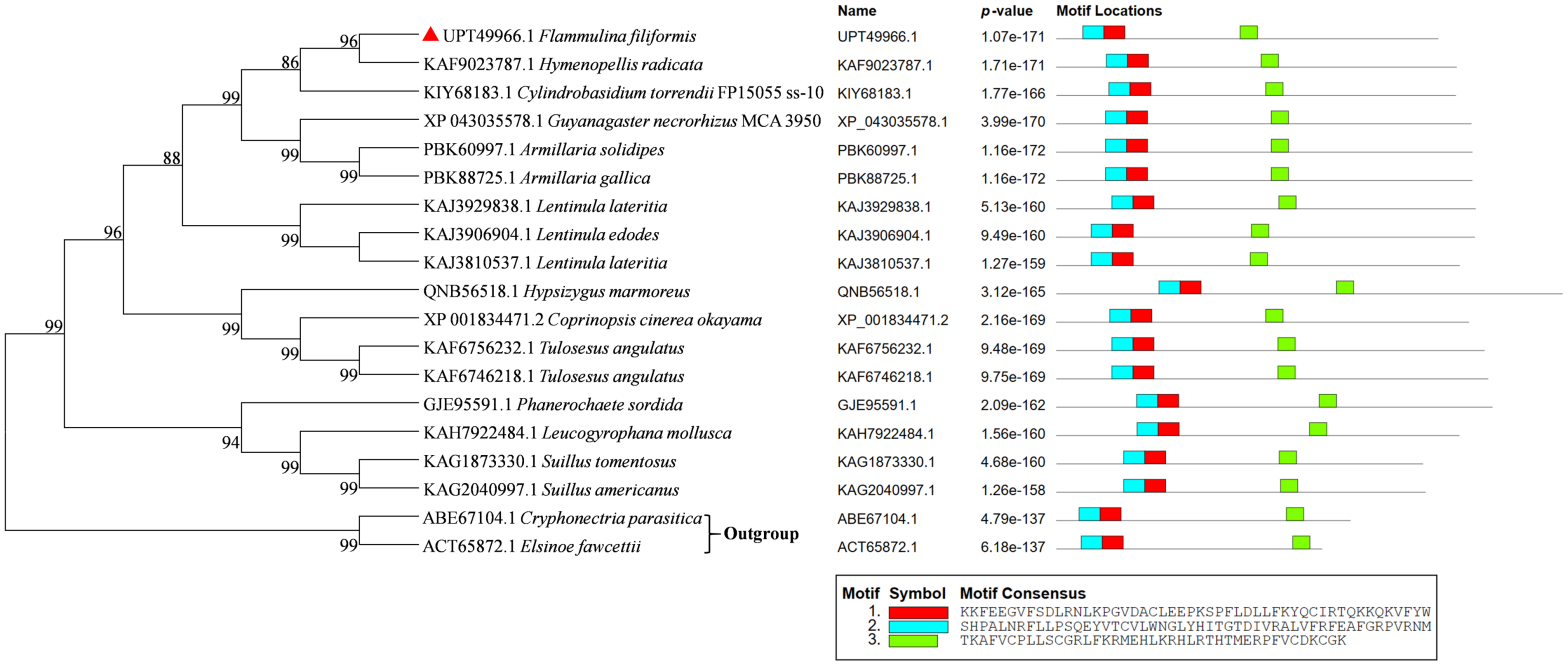
Phylogenetic tree analysis of Ste12-like from different fungi and protein structure alignment. GenBank accession numbers were indicated in front of species names. *F. filiformis* Ste12-like (GenBank accession No. UPT49966.1) was marked in red triangle. The *p*-value is defined as the probability that a random sequence (with the same length and conforming to the background) would have position *p*-value’s such that the product is smaller or equal to the value calculated for the sequence under test. Each block in the motif sites shows the position and strength of a motif site (blue and red block: Ste12-like; green block: zinc finger C2H2-type).

The phylogenetic tree of Ste12-like amino acid sequences of *F. filiformis* and other fungi was constructed by the NJ method (Figure 2). Phylogenetic analysis showed that Ste12-like contained conserved amino acid sequences, with three typical conserved motifs, namely motif 1, motif 2, and motif 3 (Figure 2). The distribution of conserved domains in the sequence was basically the same.

### 3.2. Generation of overexpression transformants

**Figure 3:**
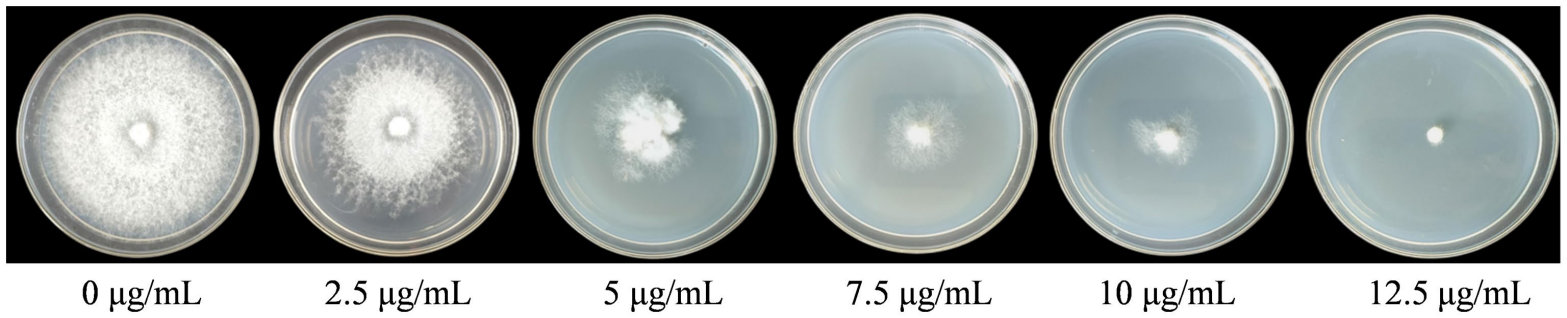
Hygromycin B sensitivity of *F. filiformis* strain FL19.

The WT strains FL19 were cultured on the PDA medium containing different concentrations of hygromycin B. The mycelia could not grow on PDA containing 12.5 μg/mL of hygromycin B (Figure 3). Therefore, the optimal screening concentration was determined to be 12.5 μg/mL.

**Figure 4:**
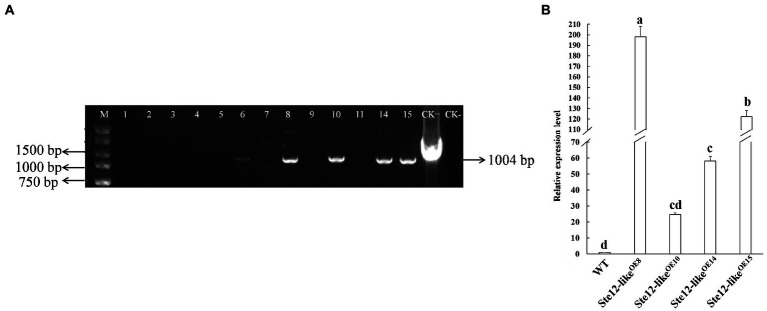
Identification of Ste12-like^OE^ transformants. **(A)** Amplified of the fragment from gene *Hpt* by PCR in putative transformants. M: Maker DL5000; 1–15: Putative Ste12-like^OE^ transformants; CK+: Positive control (*ste12-like* overexpression plasmid); CK-: negative control (wild-type: FL19). **(B)** The relative expression level of gene *ste12-like* in *F. filiformis* transformants. WT: Wild-type: FL19. Different letters indicate significant differences among strains at a *p*-value of  < 0.05 level.

Putative Ste12-like^OE^ transformants were screened by PCR with Hpt-F/R primer pairs (Table 1). The results from PCR assays showed that the gene *Hpt* fragments were inserted in the WT strain (Figure 4A). Transformants were confirmed by qRT-PCR analysis to check the expression of gene *ste12-like* (Figure 4B). The transcript levels of *ste12-like* in the Ste12-like^OE8^, Ste12-like^OE10^, Ste12-like^OE14^, and Ste12-like^OE15^ transformants were up-regulated with an approximate fold increase of 195-, 25-, 60-, and 120-fold higher than the wild-type strain FL19, respectively (Figure 4B).

### 3.3. Overexpression of gene *ste12-like* abiotic stress tolerance in *Flammulina filiformis*

**Figure 5:**
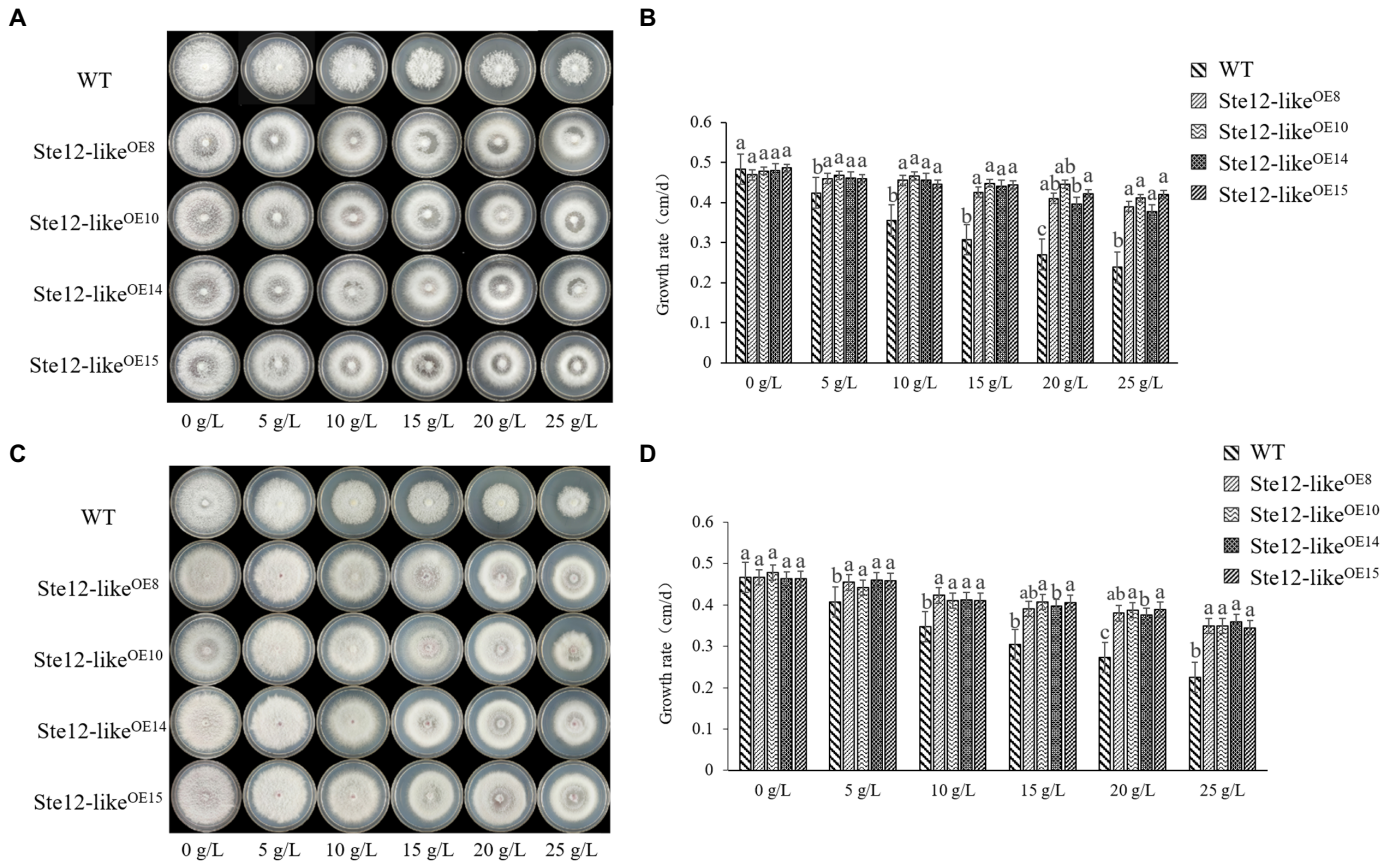
Comparison of wild-type and Ste12-like^OE^ transformants in salt stress. **(A)** Colony morphology of wild-type and Ste12-like^OE^ transformants in NaCl stress on the eighth day. **(B)** The growth rate of wild-type and Ste12-like^OE^ transformants in NaCl stress. **(C)** Colony morphology of wild-type and Ste12-like^OE^ transformants in KCl stress on the eighth day. **(D)** The growth rate of wild-type and Ste12-like^OE^ transformants in KCl stress. WT: wild-type FL19. Different letters indicate significant differences among strains at a *p*-value of  < 0.05 level.

The colony edges of Ste12-like^OE^ transformants were smoother than the WT strains while culturing on the NaCl plates (Figure 5A). When the transformants were cultured on the NaCl plates, the lack of aerial mycelia led to the formation of transparent circles in the colonies (Figure 5A). The mycelia of transformants were more resistant to stress than the WT strains on the KCl plates (Figure 5C). With increasing salt concentration, there were more aerial mycelia of transformants, while the growth rate of WT mycelia was significantly inhibited (Figures 5B,D).

**Figure 6:**
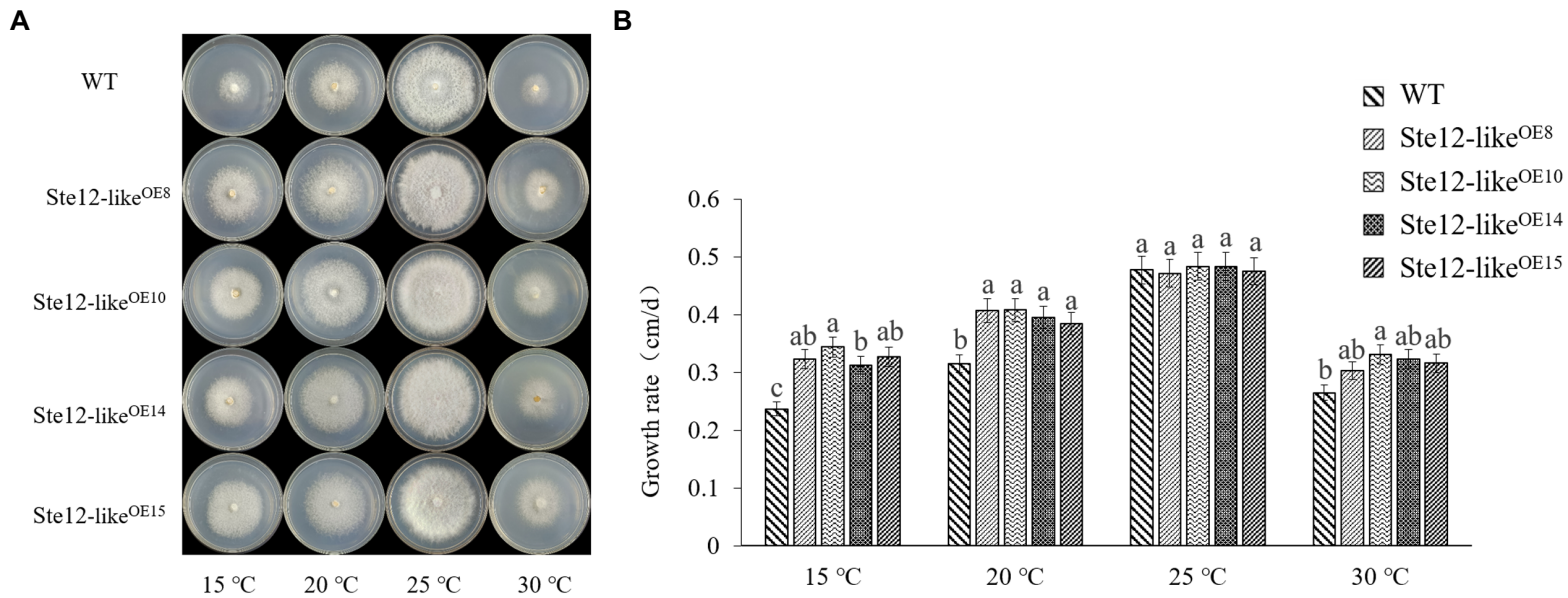
Comparison of wild-type and Ste12-like^OE^ transformants in different temperatures. **(A)** Colony morphology of wild-type and Ste12-like^OE^ transformants in different temperatures on the eighth day. **(B)** The growth rate of wild-type and Ste12-like^OE^ transformants in different temperatures. WT: wild-type FL19. Different letters indicate significant differences among strains at a *p*-value of  < 0.05 level.

There was no significant difference in mycelial morphology and growth rate between Ste12-like^OE^ transformants and wild-type strains at the optimal temperature of 25°C. However, with a decrease in temperature, the growth rate of Ste12-like^OE^ transformants was higher than wild-type strain FL19 (Figure 6). The results suggested that the Ste12-like^OE^ transformants improved the ability to resist cold stress.

**Figure 7:**
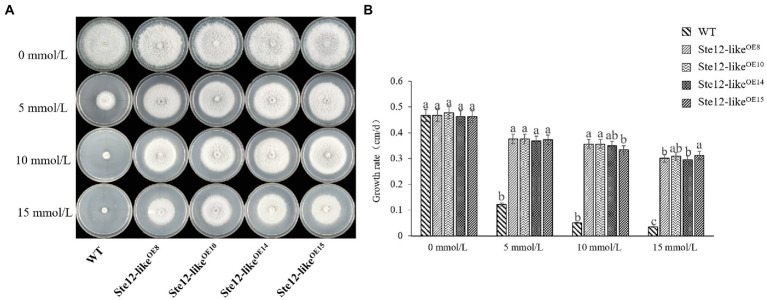
Comparison of wild-type and Ste12-like^OE^ transformants in oxidative stress. **(A)** Colony morphology of wild-type and Ste12-like^OE^ transformants in oxidative stress on the eighth day. **(B)** The growth rate of wild-type and Ste12-like^OE^ transformants in oxidative stress. WT: wild-type FL19. Different letters indicate significant differences among strains at a *p*-value of  < 0.05 level.

With increasing H_2_O_2_ concentration, the mycelium growth rate of wild-type FL19 was significantly inhibited (Figure 7). The mycelium growth rate of transformants was significantly higher than the wild-type when treated with the same H_2_O_2_ concentration. The results showed that the Ste12-like^OE^ transformants enhance oxidative stress tolerance.

### 3.4. Gene *ste12-like* regulates the fruiting body development

**Figure 8:**
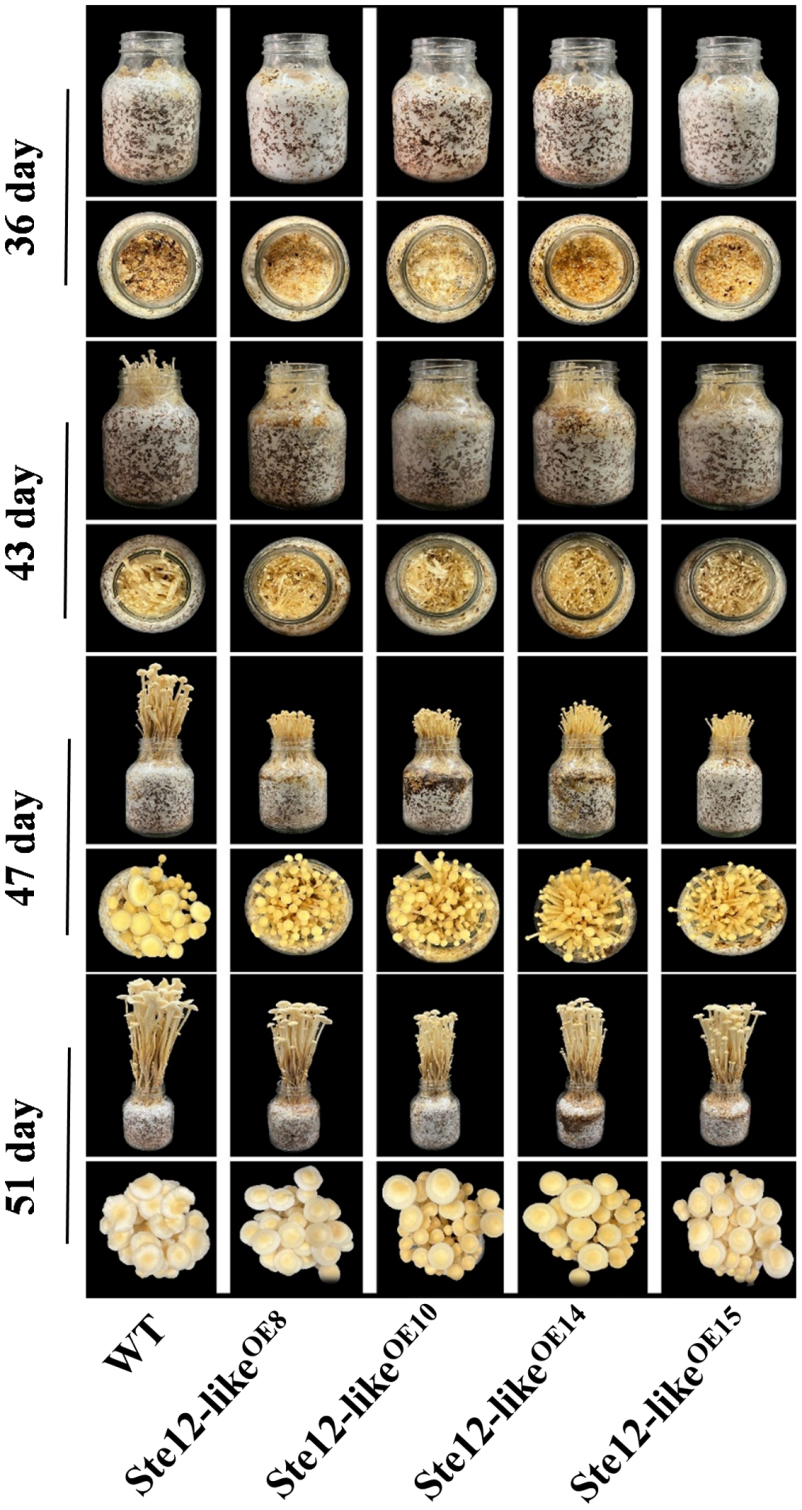
Fruiting bodies in the wild-type strain and Ste12-like^OE^ transformants.

**Figure 9:**
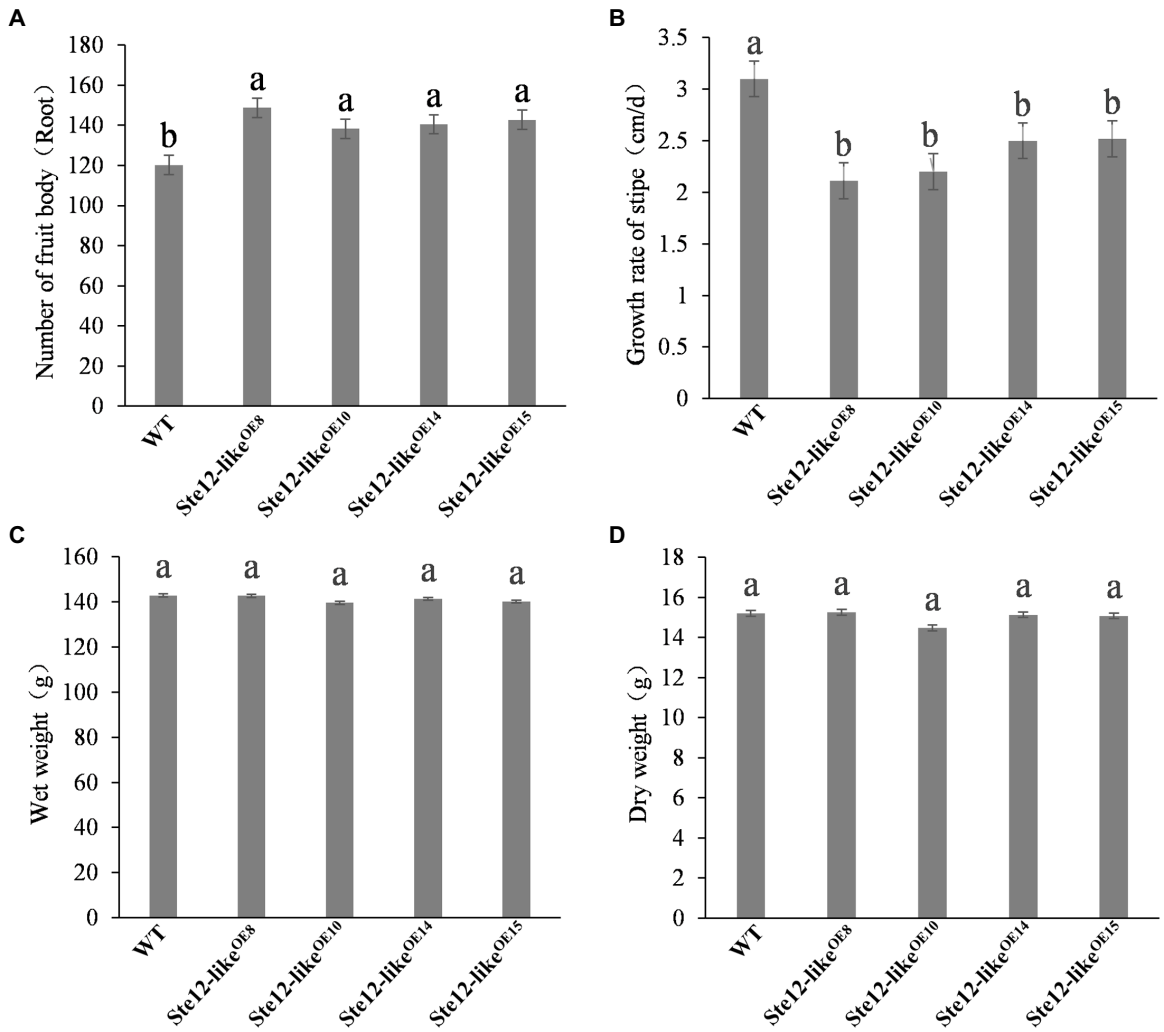
Fruiting body traits of the wild-type strain and Ste12-like^OE^ transformants. **(A)** Number of fruit bodies; **(B)** growth rate of stipe; **(C)** wet weight; **(D)** dry weight. Different letters indicate significant differences among strains at a *p*-value of  < 0.05 level.

Cultivation of the fruiting body was performed on overexpression transformants and wild-type strains separately. On the seventh day with the reference to primordium formation, normal fruiting bodies were developed in wild-type and Ste12-like^OE^ transformants (Figure 8). Although the weight of fruiting bodies was not significantly different, the overexpression mutants grew slower and had shorter stipes than the wild-type strain (Figure 9). In addition, more fruiting bodies were generated in transformants than in wild-type strains. The results presented here suggested that *ste12-like* is a regulator for the fruiting body development of *F. filiformis*.

## 4. Discussion

Sensing and responding to stress are required for fungal survival. Mammals have two MAPK pathways—p38 and Jun N-terminal kinase (JNK)—to relay stress-related signals that control cellular survival, differentiation, and apoptosis. Similarly, fungi have sophisticated signaling cascades to sense and respond to different types of stress including osmotic shock, temperature, high salt, UV irradiation, oxidative or nitrosative damage, and exposure to antifungal drugs. In fungi, Hog1 is the most extensively studied stress-activated MAPK, homologous to mammalian p38 MAPK (Bahn et al., 2007). Hog1 is also the calcineurin signaling cascade (Feng et al., 2021). The pheromone signaling pathway is a part of the MAPK signaling pathway (Tatjer et al., 2016; Li et al., 2017; Deng and Lin, 2018). Ste12 is located downstream of the pheromone signaling pathway which can be activated by mating type *B* genes (Brown and Casselton, 2001).

In *S. cerevisiae*, Ste12 mediates the transcriptional induction of cell type-specific genes in response to pheromones (Errede and Ammerer, 1989). *A. nidulans* steA (sterile12-like) is required for sexual reproduction (Vallim et al., 2000). STE12 homolog (MST12) in *M*. o*ryzae* (rice blast fungus) may regulate genes involved in infectious mycelium growth and in the expression of the cell surface sensor MSB2 (Park et al., 2002; Liu et al., 2011). Ste12 and Ste12-like proteins are significant fungal transcription factors in regulating development and pathogenicity (Hoi and Dumas, 2010). Znf domains are relatively small protein motifs that contain multiple finger-like protrusions that make tandem contacts with their target molecule (Klug, 1999). C2H2-type (classical) Znfs are the first class to be characterized. C2H2 Znfs can be divided into three groups based on the number and pattern of fingers: triple-C2H2 (binds single ligand), multiple-adjacent-C2H2 (binds multiple ligands), and separated paired-C2H2 (Iuchi, 2001). C2H2 Znfs are the most common DNA-binding motifs found in eukaryotic transcription factors and have also been identified in prokaryotes (Bouhouche et al., 2000). C2H2 Znf proteins are one of the largest and most conserved transcription factor families in the eukaryotic kingdom. It has been demonstrated that C2H2-ZFs participate in the fruiting body formation in *A. nidulans* (Kim et al., 2009), the production of oospores and swimming zoospores in *Phytophthora sojae* (Wang et al., 2009), the primordia formation in *S. commune* (Ohm et al., 2011), the yield of *Agaricus bisporus* (Pelkmans et al., 2016), hyphal growth and microsclerotia formation in *Verticillium dahlia* (Tian et al., 2017), and so on in fungi. Phylogenetic trees were constructed by screening the homologous protein of Ste12-like in GenBank using NJ analyses (Figure 2). The Ste12-like of *F. filiformis* was highly similar to species belonging to Agaricales.

*Agrobacterium*-mediated transformation was successfully used to obtain *F. filiformis* transformants with T-DNA (T-strands), which were integrated into the host genome randomly. In other studies, transformants always showed expression level variability of the target gene (Tao et al., 2019; Wu et al., 2019; Lyu et al., 2021; Meng et al., 2021). The reason for this difference may be different insertion copy numbers and the following reasons. However, certain T-DNA integration characteristics often relate to the extent of transgene expression. Multiple T-DNA copies may link at each locus. RB-to-LB linkages (head-to-tail) indicate tandem integrated T-DNAs in a direct repeat orientation, but inverted repeat LB-to-LB (tail-to-tail) or RB-to-RB (head-to-head) integration events may also occur. Head-to-head inverted repeats are common and are often associated with transgene silencing (Gelvin, 2017). In our study, four Ste12-like^OE^ transformants were obtained by *Agrobacterium*-mediated transformation. The transcript levels of *ste12-like* in the Ste12-like^OE8^, Ste12-like^OE10^, Ste12-like^OE14^, and Ste12-like^OE15^ transformants were increased 195-, 25-, 60-, and 120-fold, respectively, compared to the wild-type. Because the T-DNA was randomly integrated into the host genome, we conjecture that the expression levels might be dependent of the site (active region or inactive region) of the genome.

Transcription factor Ste12-like was a C2H2 Znf domain-containing protein. In edible mushroom *P. ostreatus*, the expression levels of the members of C2H2 Znf transcription factors are changed suddenly under heat and cold stress, suggesting that these genes may participate in abiotic stress responses (Ding et al., 2022). The *STE12α* gene of *Cryptococcus neoformans* encodes a protein containing both homeodomain and zinc finger regions; mutations in the Znfs region resulted in decreased virulence (Chang et al., 2004). In this study, the overexpression of *ste12-like* also enhanced the tolerance of salt stress, cold stress, and oxidative stress in edible mushroom *F. filiformis*. This suggested that gene *ste12-like* could play a variety of roles in response to various stresses.

The fruiting body formation in Agaricomycetes represents the most complex and unclear process in fungi. Several transcription factors (TFs) play a critical role in regulating the developmental processes of fungi (Ohm et al., 2011). The Pcc1 protein is a key element in a pathway(s) leading to pseudoclamp development and fruiting (Murata and Kamada, 2009). TFs, Bri1 and Hom1, of the model fungus *S. commune* are involved in the late stages of mushroom development, while Wc-2, Hom2, and Fst4 function early in development (Pelkmans et al., 2017). However, studies on TFs in the fruiting body development of mushroom-forming species are still at the initial stage.

We have measured the expression of gene *ste12-like* in different tissues at different development stages of the fruiting body by both RNA-seq and quantitative real-time PCR in the previous study (Liu et al., 2022). The expression of gene *ste12-like* was down-regulated in elongating stipe. It suggested that the overexpression of *ste12-like* might inhibit the elongation of the stipe. The fruiting experiment of four transformants, which were obtained in this study, also confirmed this conclusion. The growth rate and length of a stipe in overexpression transformants were obviously lower than those of wild-type strains (Figures 8,9). It is reported that the pheromone signaling pathway downstream transcription factor Ste12 might play important roles in fruiting body formation and development (Hoi and Dumas, 2010; Chen et al., 2020). However, the regulatory mechanism of Ste12-like involved in these processes is still unclear. Therefore, the downstream target genes of transcription factor Ste12-like should be studied further in order to illustrate the regulation molecular mechanism in abiotic stress tolerance and fruiting body development.

## Data availability statement

The datasets presented in this study can be found in online repositories. The names of the repository/repositories and accession number(s) can be found in the article/Supplementary material.

## Author contributions

WW designed this study. XL and QW performed the experiments. AL, FL, LM, PW, and YZ analyzed all data. ZL, LW, and WW wrote the initial manuscript. All authors contributed to the writing and editing of the final manuscript. All authors have read and agreed to the published version of the manuscript.

## Funding

This research was supported by the National Natural Science Foundation of China (Nos. 31902086; 32002108) and the Mushroom Technology System of Shandong Province (No. SDAIT-07-06).

## Conflict of interest

The authors declare that the research was conducted in the absence of any commercial or financial relationships that could be construed as a potential conflict of interest.

## Publisher’s note

All claims expressed in this article are solely those of the authors and do not necessarily represent those of their affiliated organizations, or those of the publisher, the editors and the reviewers. Any product that may be evaluated in this article, or claim that may be made by its manufacturer, is not guaranteed or endorsed by the publisher.

## Footnotes

^1^ http://psort.hgc.jp/form2.html

^2^ http://www.ebi.ac.uk/interpro/

^3^ http://www.ebi.ac.uk/Tools/msa/clustalo/

^4^ https://meme-suite.org/meme/tools/meme
