# Supplementary material for: The transcription factor Ste12-like increases the mycelial abiotic stress tolerance and regulates the fruiting body development of Flammulina filiformis
Source: Front Microbiol. 2023 May 4;14:1139679. doi: 10.3389/fmicb.2023.1139679 (PMC10192742; doi:10.3389/fmicb.2023.1139679)
Supplement: Supplementary file 1 [file Data_Sheet_1.pdf]

Supplementary Table 1. The NLS positions of *Flammulina filiformis* Ste12-like and homologous proteins.

Scientific Name	Accession	NLS positions and sequences	Number of NLS
Cryphonectria parasitica	ABE67104.1	403-411 (PTYKQRRRR)	1
Elsinoe fawcettii	ACT65872.1	426-434 (PTYKQRRRR)	1
Hypsizygus marmoreus	QNB56518.1	494-497 (RKKK); 568-571 (KKPR); 703-709 (PQCKKRF); 997-1003 (PIRRHRS)	4
Coprinopsis cinerea okayama	XP_001834471.2	323-326 (RRPR); 349-354 (KHRKR); 447-450 (RKPR); 793-799 (PIRRHRS)	4
Tulosesus angulatus	KAE6756232.1	373-376 (RRKK); 466-469 (RKPR); 564-570 (PKCKKRF); 816-822 (PIRRHRS)	4
Tulosesus angulatus	KAF6746218.1	373-376 (RRKK); 466-469 (RKPR); 564-570 (PKCKKRF); 824-830 (PIRRHRS)	4
Lentinula lateritia	KAJ3929838.1	346-355 (PTYKQRRKKP); 796-802 (PVRRHRS)	2
Lentinula edodes	KAJ3906904.1	309-318 (PTYKQRRKKP); 791-797 (PVRRHRS)	2
Lentinula lateritia	KAJ3810537.1	309-318 (PTYKQRRKKP); 756-762 (PVRRHRS)	2
Leucogyrophana mollusca	KAH7922484.1	438-447 (PTYKQRRKKP); 508-511 (RRKK); 825-831 (PLRRHRS)	3
Suillus tomentosus	KAG1873330.1	391-400 (PTYKQRRKKP); 745-751 (PHRRHRS)	2
Suillus americanus	KAG2040997.1	395-404 (PTYKQRRKKP); 453-459 (PSVRRAK); 748-754 (PHRRHRS)	3
Phanerochaete sordida	GJE95591.1	428-436 (PTYKQRRKK); 862-868 (PIRRHRS)	2
Cylindrobasidium torrendii FP15055 ss-10	KIY68183.1	359-367 (PTYKQRRKK); 744-750 (PSRRHRS)	2
Guyanagaster necrorhizus MCA 3950	XP_043035578.1	366-374 (PTYKQRRKK); 801-804 (RRHR)	2
Armillaria solidipes	PBK60997.1	366-374 (PTYKQRRKK); 802-805 (RRHR)	2
Armillaria gallica	PBK88725.1	366-374 (PTYKQRRKK); 802-805 (RRHR)	2
Flammulina filiformis	UPT49966.1	298-306 (PTYKQRRKK); 714-720 (PVRRHRS)	2
Hymenopellis radicata	KAF9023787.1	352-360 (PTYKQRRKK); 765-771 (PVRRHRS)	2

Through the analysis of the NLS position of *F. filiformis* and homologous proteins, it was found that the NLS sequence of *F. filiformis* was consistent with *H. radicata*, and their genetic relationship was the closest. Two outgroup sequences have only one NLS. The sequence of NLS of distantly related species is similar, but not very consistent, and the number is also different.
