# Supplementary figures and images for: The transcription factor Ste12-like increases the mycelial abiotic stress tolerance and regulates the fruiting body development of Flammulina filiformis
Source: Front Microbiol. 2023 May 4;14:1139679. doi: 10.3389/fmicb.2023.1139679 (PMC10192742; doi:10.3389/fmicb.2023.1139679)

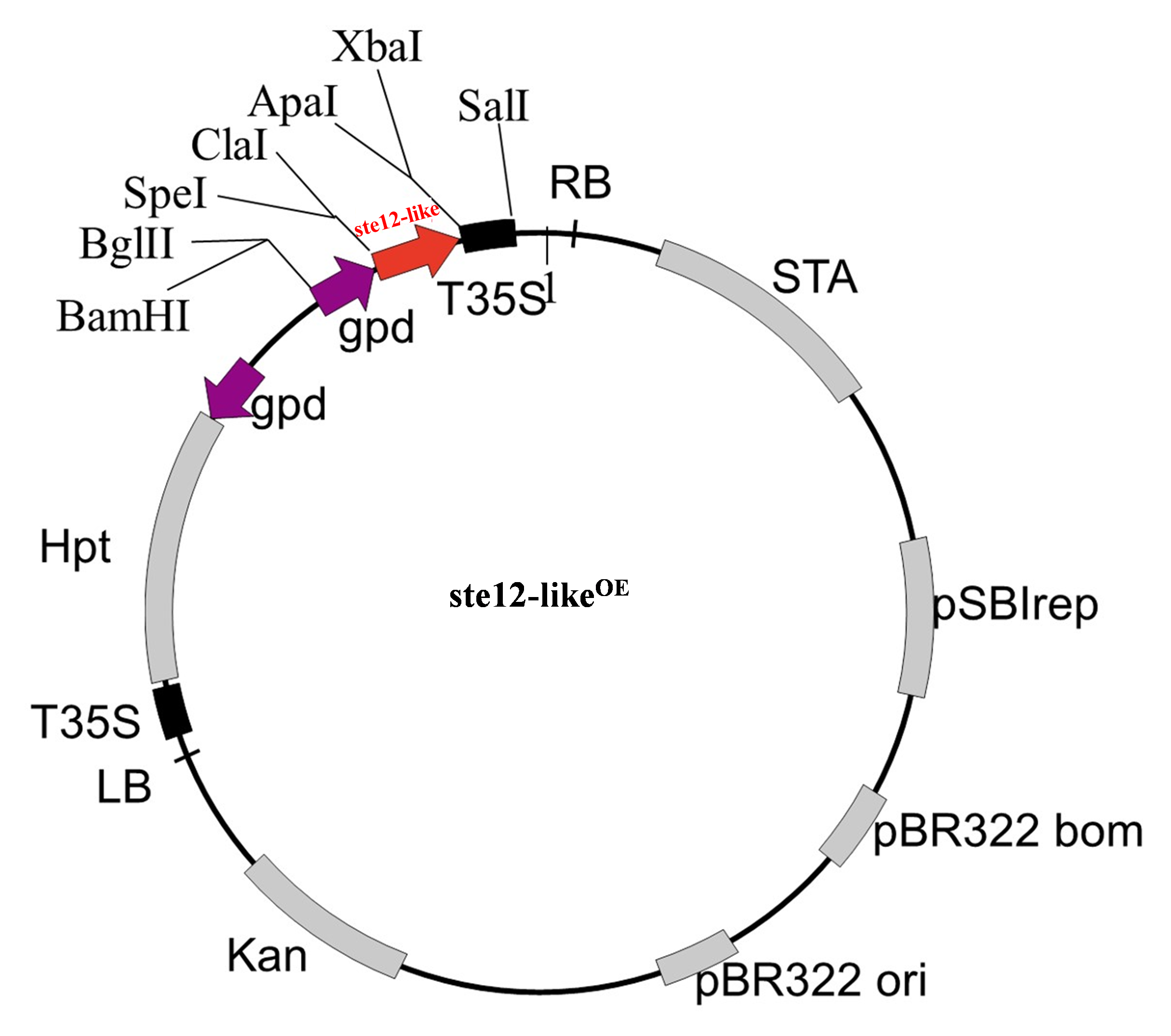

Supplement: Supplementary file 2 [file Image_1.TIF]
